# Asparaginase-associated Pancreatitis Complicated by Pancreatic Fluid Collection Treated with Endoscopic Cistogastrostomy in Pediatric Acute Lymphoblastic Leukemia: A Case Report and Systematic Review of the Literature.

**DOI:** 10.46989/001c.90958

**Published:** 2023-12-20

**Authors:** Giulia Fiumana, Alessia Pancaldi, Helga Bertani, Valentina Boarino, Monica Cellini, Lorenzo Iughetti

**Affiliations:** 1 Post Graduate School of Pediatrics, Department of Medical and Surgical Sciences of the Mothers, Children, and Adults University of Modena and Reggio Emilia; 2 Pediatric Hematology Oncology Unit Policlinico di Modena; 3 Gastroenterology and Endoscopy Unit Policlinico di Modena; 4 Gastroenterology Unit Policlinico di Modena; 5 Post Graduate School of Pediatrics, Department of Medical and Surgical Sciences of the Mothers, Children, and Adults University of Modena and Reggio Emilia, Italy; 6 Pediatric Hematology Oncology Unit Azienda Ospedaliero Universitaria Policlinico di Modena, Italy

**Keywords:** Asparaginase, Pancreatitis, Pancreatic Fluid Collection, Gastrointestinal Endoscopy, Lymphoid Leukemia, Pediatric Oncology

## Abstract

Asparaginase-associated pancreatitis complicates 2-10% of patients treated for acute lymphoblastic leukemia, causing morbidity and discontinuation of asparaginase administration. Among acute complications, pancreatic fluid collections can be managed conservatively, but intervention is indicated when associated with persistent insulin therapy need and recurrent abdominal pain. Endoscopic treatment has become the standard approach in adult patients, with increasing favorable evidence in children. This work compares the characteristics of a pediatric oncology patient treated at our institution with reported literature experiences, showing feasibility, safety and effectiveness of endoscopic approach.

## 1. Introduction

Acute lymphoblastic leukemia (ALL) is the most common tumor in children, accounting for an estimated annual incidence of more than 5000 cases worldwide.^1^ The outcome has dramatically increased over the past decades, reaching a 5-year survival rate of over 80% in high-income countries.^2^ This success is the result of therapeutic protocol improvement through the cooperation between research groups worldwide; however, new clinical trials are currently still based on essential drugs that started being used in the ’50-’60s, such as methotrexate, 6-mercaptopurine, vincristine, and asparaginase.^3^ Asparaginase was discovered in 1950 after the observation that lymphomas in rats and mice regressed administering guinea pig serum.^4^ That effect lies in the enzyme L-asparaginase amidohydrolase that deamidates asparagine into aspartic acid and ammonia, depleting cellular levels of asparagine, considered an essential amino acid for protein synthesis in leukemic cells (while normal cells can synthesize asparagine from aspartate, leukemic cells depend on exogenous sources of asparagine for survival).^5^

Preparations used are native *Escherichia coli* (*E. coli*) asparaginase, polyethylene glycolate (PEG)-asparaginase, and Erwinia asparaginase.

The native form of L-asparaginase amidohydrolase derives from *E. coli* and has been dismissed because it was limited by its short half-life (needing to be administered frequently), and high immunogenicity, resulting in hypersensitivity reactions or silent inactivation.^6–8^

PEG-asparaginase is the pegylated form of *E. coli* L-asparaginase. Thanks to the increased circulation time of the enzyme and reduced immunogenicity, PEG-asparaginase widely replaced the native form. The conjugation of monomethoxypolyethylene glycol and L-asparaginase is usually made with the succinimidyl succinate (SS-PEG) molecule. However, a new product consisting of a succinimidyl carbonate (SC-PEG) linkage (calaspargase, CALASP), seems to provide no significant differences in treatment outcomes or toxicities with a longer serum activity, allowing a lengthening time interval between doses.^6,7^ Calaspargase is approved for the pediatric population exclusively in the USA.^8^

Erwinia asparaginase (pegcrisantaspase) has a unique immunogenic profile without cross-reactivity with the *E. coli*-derived product, because of a different bacterial origin (*Erwinia chrysanthemi*). It is used after hypersensitivity reactions to *E. coli*–derived asparaginases, with higher dosage at a greater frequency due to its shorter half-life.^6,7^

Current clinical trials implemented asparaginase management using Therapeutic Drug Monitoring (TDM) through serum asparaginase activity (SAA) to investigate the response to therapy, detect neutralizing antibodies in hypersensitivity reactions or silent inactivation, and define individualized drug dosage.^8^

Adverse effects related to asparaginase therapy are hypersensitivity reactions, hepatotoxicity, hypertriglyceridemia, hyperglycemia, pancreatitis, encephalopathies, and thrombotic or bleeding complications.^9^

Asparaginase-associated pancreatitis (AAP) occurs with a reported incidence of up to 18%.^10–15^ The mortality is low, but significant acute morbidity and chronic complications cause APP to be a leading cause of asparaginase discontinuation, this being possibly associated with an increased risk of relapse, given the core importance of asparaginase in ALL treatment.^13,16,17^

APP is defined by the presence of at least two between: abdominal pain strongly suggestive of pancreatitis, serum lipase or amylase three or more times the upper normal limits (UNL), and characteristic imaging findings of pancreatitis (ultrasound, CT, or MRI). Mild APP is defined by the persistence of symptoms and enzyme elevations more than three times UNL for less than 72 h; when they last more than 72 h or complications such as hemorrhagic pancreatitis, pancreatic abscess, or fluid collection occur, APP is defined as severe.^18^

After a severe APP, asparaginase is usually discontinued. Milder cases can benefit from ASP re-exposure, even if these patients face a risk of APP recurrence up to 50%, as subsequent episodes seem less severe and not associated with an increased risk of developing persisting complications.^13^

The pathogenesis is unclear, probably related to asparagine depletion leading to reduced protein synthesis.^19^ Still, possible risk factors are older age, higher ALL risk stratification, genetic polymorphisms (CNOT3, ULK2, RGS6, HOGA1, CPA2, ADAMTS17, MBP1A, SPECC1, CFTR, ASNS, GRIA1, HLA-DRB1, NFATC2, IL16, SPEF2, SOD2, ATF5) and severe hypertriglyceridemia.^19–24^ Some studies found a relationship with higher asparaginase doses.^12,19,25^ In contrast, others noted that most APP cases develop early after the first few doses, suggesting a role in pancreatitis predisposition rather than a cumulative drug effect.^10,26^

APP treatment is primarily supportive, based on fluid replacement, pain relief, and monitoring the development of complications.^19,24,27^ Bowel rest is indicated in the acute phase, but early enteral feeding reduces complications in adults’ non-asparaginase-related pancreatitis.^28^ Broad-spectrum antibiotics should be given in severe cases suspected of sepsis.^19^ Some studies showed a role for agents such as octreotide (somatostatin analog) in preventing AAP, by reducing inflammation through its capacity to inhibit the secretion of pancreatic enzymes,^29^ or galactose.^30^ Finally, successful results were observed with continuous regional arterial infusion of protease inhibitor and antibiotic.^31^

Complications following APP can be acute (pleural effusions, multiorgan failure, death), subacute (pancreatic necrosis, infection, formation of pancreatic fluid collections), or chronic (persistent need of insulin and chronic abdominal pain).^19,24^

Pancreatic fluid collections (PFC) are classified based on duration and presence of necrosis as acute peripancreatic fluid collections (<4 weeks, no necrosis), acute necrotic collections (<4 weeks, necrosis), pseudocysts (>4 weeks, no necrosis), and walled-off necrosis (WON, >4 weeks, necrosis).^32^ The management can be conservative or operative through percutaneous external drainage, endoscopic internal drainage, or surgical drainage. Indications for intervention in adult patients are historically based on the size of PFC and presence of symptoms.^33–35^ The pediatric population seems to benefit from a more conservative approach, because of a higher spontaneous resolution regardless of the size, limiting operative treatment to symptomatic patients.^27,36–39^

When the decision for intervention is made, endoscopic treatment is recommended in adults as the first choice approach, because of minimal invasion and lower morbidity, despite a seemingly higher recurrence rate, compared to surgical drainage.^34,35,40^ Reported cases in children show similar good safety and efficacy of endoscopic cystogastrostomy.^27,41–48^

It has been noted that the occurrence of PFC is more frequent after AAP (up to 1 in 4 cases)^13,49^ than in other etiologies. Therefore, it is important to recognize this complication and optimize treatment to reduce morbidity and delay in chemotherapy.

## 2. Case presentation

**Figure 1.**
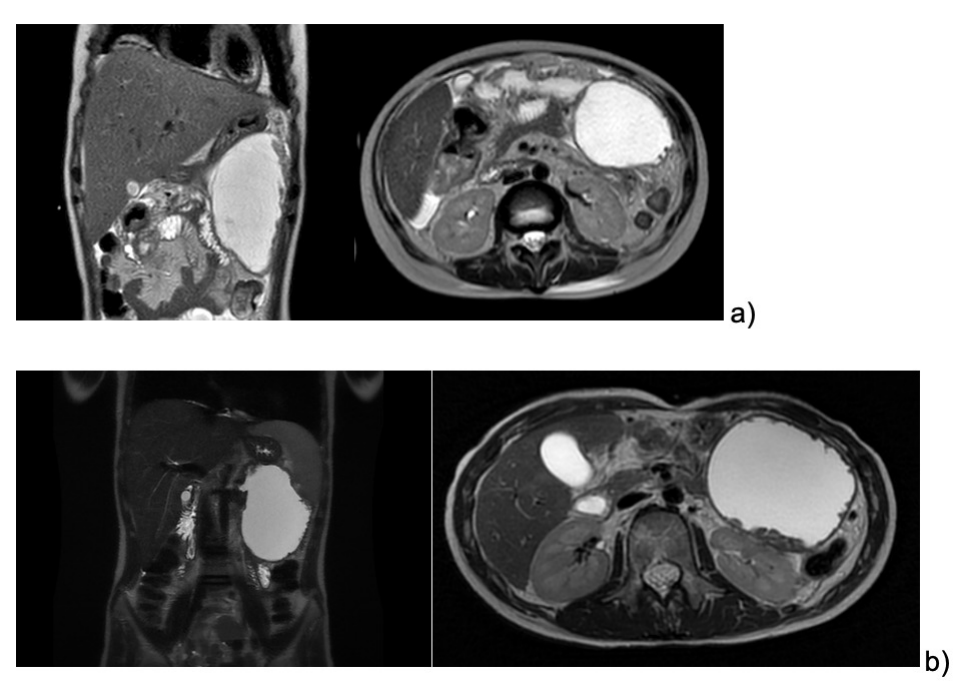
a) Abdominal MRI 14 days after APP; b) Cholangio MRI 3 months after APP

We describe the case of an 11 year-old female patient affected by pre-B ALL treated according to the early non-high-risk group in AIEOP-BFM-2017 protocol. One week after the start of Protocol Ia, a staging brain MRI showed cerebral venous thrombosis, which was treated with intravenous heparin for three weeks, followed by subcutaneous injections. The patient received two doses of PEG-asparaginases on days +12 and +26, and on day +48 she presented at the Emergency Department complaining of epigastric abdominal pain worsening after meals. Blood tests showed increased pancreatic enzymes (lipase 1383 U/l, amylase 310 U/l) and coagulation alterations (PT ratio 1,22, aPTT 1,09, Fibrynogen 111 mg/dl, Antithrombin 31%, D-Dimer 1087) suggesting acute pancreatitis, which was confirmed by a CT scan showing enlarged pancreas volume with multiple hypointense areas of necrotizing pancreatitis associated with local thrombosis of the distal splenic vein, and peritoneal and pleural effusions. A nasogastric tube was positioned and fasting started. After a few hours, her general conditions precipitated into a septic-like shock state, requiring fluid boluses, albumin, fibrinogen, antithrombin III, and broad-spectrum empirical antibiotics. Echocardiography showed mild pericardial effusion and signs of myocardial injury. The clinical and laboratory manifestations progressively resolved during the following days. After two weeks, an abdominal MRI revealed the initial formation of a non-encapsulated pancreatic fluid collection along the left side and hypochondrium, measuring 13 x 7 x 7 cm (Fig.1a).

The patient was discharged on oral mercaptopurine and methotrexate maintenance therapy after one month of chemotherapy protocol interruption, and proceeded with the imaging and clinical follow-up for the next three months.

**Figure 2.**
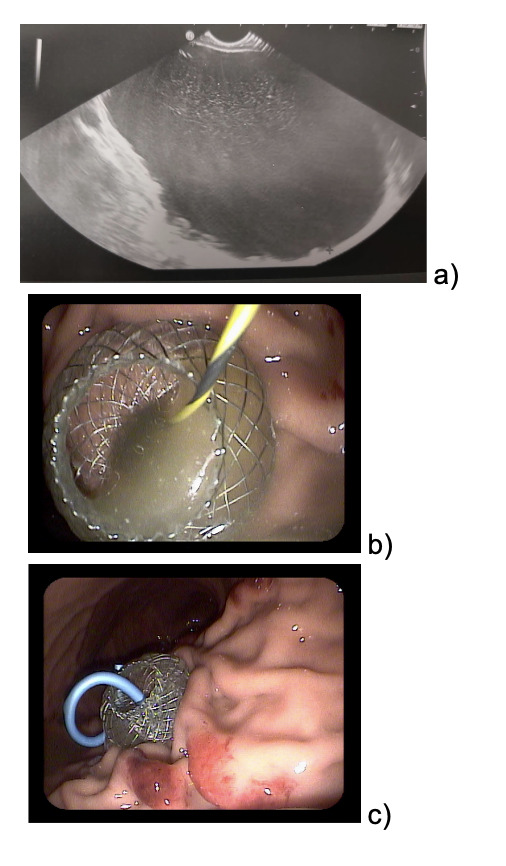
a) echoendoscopy; b-c) stent placement

**Figure 3.**
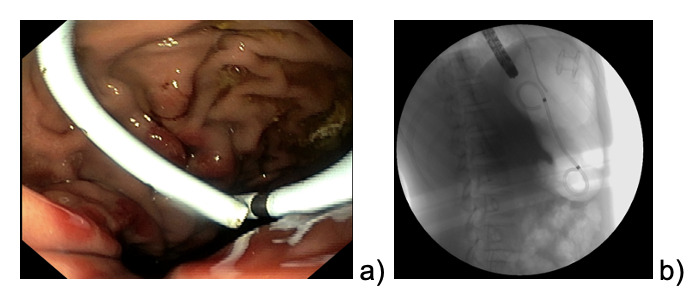
a) DPPS positioning; b) radiological control

The patient continued to refer mild abdominal pain, and a cholangio MRI confirmed the persistency of an encapsulated pancreatic fluid collection without size reduction (Fig.1b). After a multidisciplinary discussion, the decision was made to perform an endoscopic-ultrasound (EUS) guided cystogastrostomy with a 10 x 10 mm lumen-apposing metal stent (LAMS) (Hot-Axios™ system) under general anesthesia. During the procedure, after the release of the LAMS, a 7 cm x 7 Fr double-pigtail plastic stent (DPPS) was coaxially released (Fig.2); the post-operative course was uneventful and the patient was discharged after 48 hours. Seven days thereafter, an abdominal ultrasound confirmed the complete resolution of the PCF, the LAMS was removed, and a “soft” DPPS 7 cm x 10 Fr (Solus-Cook®) was positioned endoscopically in the cystic cavity under endoscopic and radiological control (Fig.3). The patient was dismissed the following day without any complications, and two weeks thereafter she restarted the AIEOP-BFM-2017 protocol at Short Consolidation B, 4 months after the interruption of the consolidation phase. No pancreatic fluid collection was observed at the following abdominal ultrasounds, the latest performed 7 months after interventions, together with an abdominal x-ray that showed the persistent presence of the double pig-tail. The prosthesis removal will be scheduled together with the central venous catheter removal at the end of the maintenance phase, due in a few months, so as to perform a single sedation.

## 3. Methods

**Figure 4.**
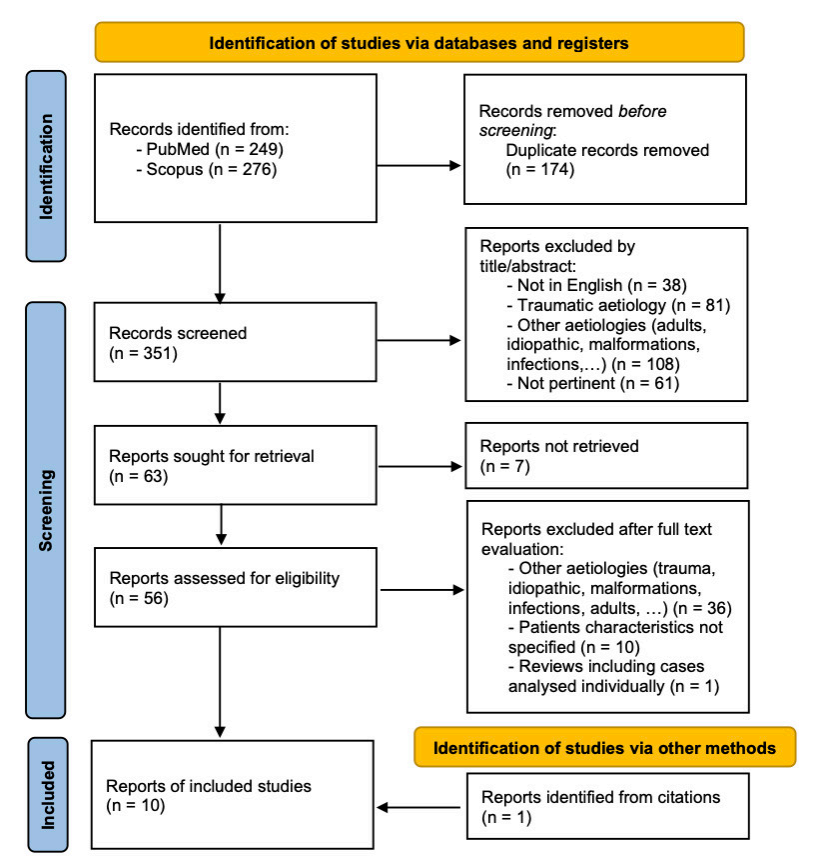
PRISMA flowchart

A systematic literature review was performed through a combined search on PubMed [using terms ((pancreatic fluid collection) OR (pancreatic pseudocyst)) AND ((endoscopy) OR (cystogastrostomy) OR (endoscopic drainage)) AND ((children) OR (pediatric))] and Scopus databases [using terms TITLE-ABS-KEY (((pancreatic AND fluid AND collection) OR (pancreatic AND pseudocyst)) AND ((endoscopy) OR (cystogastrostomy) OR (endoscopic AND drainage)) AND ((children) OR (pediatric)))]. A total of 525 records were identified on 31 May 2023, of which 174 were duplicated; through titles and abstracts screening 288 reports were excluded; of the remaining 63 reports, full text was unavailable for 7 papers. Therefore 56 reports were analyzed and 9 selected; one additional case^17^ was found through citations screening, with a total of 10 cases being included in this review. Figure 4 shows the selection process performed using PRISMA flowchart.^50^

## 4. Discussion

Through a literature review, we identified 10 studies including each a pediatric patient affected by leukemia with PFC after AAP treated endoscopically; their characteristics are reported in Table 1. Patients’ ages varied from 2 to 17 years old. Studies reporting the onset time of PFC showed that it occurred within 1 month after AAP.^51–53^ Procedures were performed within 2 months^51,53,54^ while, in our patient, the decision to postpone the treatment for a few weeks was based on her mild symptoms, no signs of infections and the possibility to maintain conservative management. EUS-guided cystogastrostomy was the first choice approach in most patients, according to ESGE Guidelines,^55,56^ while in three cases, the patients were first treated with percutaneous drainage that failed, requiring subsequent endoscopy.^17,52,57^ Some studies reported the positioning of more than one cystogastric stent^51–53^. In our patient, the decision to use a LAMS was made by the endoscopist during the procedure to rapidly improve the patient’s symptoms and to create a bigger fistula between the stomach and the PFC to enhance drainage of thick fluid; after 7 days the LAMS was removed and a plastic stent was positioned for long-term healing of the PFC. Studies reported a stent indwell time between 1 month and 8 months, shorter for LAMSs, as those stents should not be left in place for more than 4 weeks because of the risk of stent burial and bleeding.^34^ In the reviewed articles, DPPSs were left in place for up to 8 months,^51^ but cases of permanent placement were also reported.^58^

There were no complications after endoscopic treatment (apart from the isolation of bacteria, without further details, in one patient who underwent multiple procedures^17^), and the treatment was effective in all patients without the need for further interventions, except for one patient who underwent an endoscopic necrosectomy to remove residual necrotic material from pancreatitis^52^; no case of relapse was reported.

**Table 1.** Patients’ characteristics from studies selected for the literature review.

Reports	Age in years	PFC time onset after AAP	PFC measures in cm	Time from PFC diagnosis to intervention	Technique and type of stent used for cystostomy	Complications / hospital stay post intervention	Chemotherapy restart time after intervention	PFC relapse
Our patient	11	2 weeks	13 × 7 × 7	12 weeks	10x10mm LAMS and 7Frx7cm double pig-tail plastic stent coaxially, substituted with 10Frx7cm double pig-tail plastic stent after 1 week	No / 1 day	2 weeks	No (7 months follow-up)
Flores-Calderón J^11^	-	-	> 5	-	-	No / -	-	-
Yoder SM^41^	13	-	10	-	Harmonic Scalpel and figure-of-eight suture	-	-	-
Makin E^54^	8.2	-	20 × 17 × 11	9 weeks	-	- / 3 days	-	-
Musumba C^59^	10	-	20 × 14	-	10mm×2cm metal stent (NAGI stent) and nasocystic catheter for irrigation. The metal stent was removed after 12 weeks	-	12 weeks	No (15 months follow-up).
Sial GZ^51^	4	3 weeks	8.5 × 7	5 weeks	Two 7Frx7cm silicon double pig-tail stents removed after 8 months	-	-	No
Kheder J^52^	5	10 days	7.5 × 6 × 9	-	Recurrence less than 2 weeks after percutaneous drainage with 8Fr pigtail catheter treated with cystogastrostomy, placing 4 plastic double pigtail stents (1 7Fr×4cm and 3 10Fr×1cm). Endoscopic necrosectomy after 4 weeks and cystogastrostomy stents removed 3 weeks later	-	-	No (1 year follow-up)
Costa PA^57^	17	-	-	-	Recurrence after percutaneous drainage treated with cystogastrostomy, placing a 15 mm diameter AXIOS stent removed after 34 days	No	-	No
El-Gohary Y^38^ *	-	-	-	-	ERCP with pancreatic duct stent placement for 50 days	-	0 - 12.3 days (not specified)	No
Walsh LT^53^	2	3 weeks	9.8 × 7.2 × 10.2	5 weeks	Two double-pigtail stents (10Fr×10cm and 7Fr×10 cm) removed after 1 month	-	-	No (3 years follow-up)
Kuo S-H^17^ *	-	-	-	-	CT-guided drainage, laparoscopic debridement and drainage, EUS-guided cystogastrostomy drainage	Identification of Klebsiella pneumoniae, oxacillin sensitive Staphylococcus epidermidis, oxacillin resistant Staphylococcus epidermidis	-	No

*these studies reported a cystogastrostomy intervention but the patients’ characteristics were not described

## 5. Conclusions

Treatment of pancreatic fluid collections in pediatric oncological patients is not standardized, and the decisions require the multidisciplinary skills of oncologists, endoscopists/surgeons, and radiologists. The procedure showed safe and effective results from our experience. To our knowledge, this is the first review that compares all pediatric ALL patients reported to have been treated with endoscopic cystogastrostomy. Given the rarity of the condition, more data should be collaboratively collected to standardize the optimal management and avoid further complications and chemotherapy delay.

### Conflict of Interest

The authors have no conflict of interest or funding to declare.

### Ethical Standard

All procedures were followed by the ethical standards of the responsible committee on human experimentation (institutional and national) and with the Helsinki Declaration of 1975, as revised in 2008.

### Informed Consent

Parental/guardian consent was obtained.

### Authors’ Contributions per CRediT

Conceptualization: Giulia Fiumana (Equal), Alessia Pancaldi (Equal). Formal Analysis: Giulia Fiumana (Equal), Alessia Pancaldi (Equal), Helga Bertani (Equal), Valentina Boarino (Equal). Investigation: Giulia Fiumana (Equal), Alessia Pancaldi (Equal), Helga Bertani (Equal), Valentina Boarino (Equal). Writing – original draft: Giulia Fiumana (Equal), Alessia Pancaldi (Equal), Helga Bertani (Equal), Valentina Boarino (Equal). Methodology: Alessia Pancaldi (Equal). Writing – review & editing: Monica Cellini (Equal), Lorenzo Iughetti (Equal). Supervision: Monica Cellini (Equal), Lorenzo Iughetti (Equal).
